# The reconstruction of complex networks with community structure

**DOI:** 10.1038/srep17287

**Published:** 2015-12-01

**Authors:** Peng Zhang, Futian Wang, Xiang Wang, An Zeng, Jinghua Xiao

**Affiliations:** 1 School of Science, Beijing University of Posts and Telecommunications, Beijing 100876, P.R. China; 2 State Key Laboratory of Information Photonics and Optical Communications, Beijing University of Posts and Telecommunications, Beijing 100876, China; 3 School of Systems Science, Beijing Normal University, Beijing 100875, P.R. China

## Abstract

Link prediction is a fundamental problem with applications in many fields ranging from biology to computer science. In the literature, most effort has been devoted to estimate the likelihood of the existence of a link between two nodes, based on observed links and nodes’ attributes in a network. In this paper, we apply several representative link prediction methods to reconstruct the network, namely to add the missing links with high likelihood of existence back to the network. We find that all these existing methods fail to identify the links connecting different communities, resulting in a poor reproduction of the topological and dynamical properties of the true network. To solve this problem, we propose a community-based link prediction method. We find that our method has high prediction accuracy and is very effective in reconstructing the inter-community links.

Many complex systems can be naturally described by complex networks, which has largely deepened our understanding of the structure of real systems. For example, many topological properties, such as small-world^1^, scale-free^2^, assortativity^3^, community^4^ and rich club^5^, have been uncovered in not only the social and technology systems we are using everyday^6^,^7^,^8^,^9^,^10^,^11^, but also the biology systems within our bodies^12^,^13^,^14^. In addition, network representation is useful from practical point of view. It allows us to optimize the systems for higher functionality^15^,^16^,^17^ and predict the future evolution of real systems^18^,^19^. Link prediction is one of these significant research problems^20^. It aims to estimate the likelihood of the existence of a link between two nodes, based on observed links and nodes’ attributes in a network. With this problem solved, a large amount of cost in lab experiment for identifying the missing data could be reduced^20^.

Link prediction methods assume that similar nodes are those that have similar connectivity patterns. Therefore, the essential problem in link prediction is to objectively estimate the similarity between nodes^21^. Up to now, many similarity metrics on link prediction have been proposed. The most straightforward method is the so-called Common Neighbor index which directly computes the number of overlapped neighbors between two nodes to determine their similarity^22^. This index, though simple, has many shortcomings. It is strongly biased to the large degree nodes and it works poorly in sparse networks. To solve these problems, many other methods, such as Jaccard^23^, Resource Allocation^24^, Local Path methods^25^ etc, are designed. Recently, some attention has also been paid to study link prediction in weighted^26^,^27^, directed^28^,^29^, bipartite^30^,^31^ networks. Moreover, some link prediction methods have been introduced to detect the spurious connections in complex networks^32^.

In order to quantify the quality of link prediction, the index called *area under the receiver operating characteristic curve (AUC)* is usually used^33^. In practice, it calculates the probability that a true link has a higher link prediction score than a nonexisting link. In the case of predicting missing links, the predicted links need to be added to the observed networks to obtain the reconstructed networks^20^. The 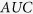 index can only reflect the fraction of corrected links added to the network, but cannot capture whether the reconstructed network has the same or similar structural and dynamical properties as the true network. This is especially important in the networks with community structure^34^. It can happen in such networks that a link prediction method correctly identifies many missing links, but completely neglects those links connecting different communities. These inter-community links actually play an important role in the networks. They characterize the interactions between different clusters^35^. They are also strongly related to many global network properties such as average shortest path and the betweenness centrality^36^. Without these links, some dynamical properties such as bond percolation will be largely distorted^37^.

In this paper, we apply several representative link prediction methods to reconstruct complex networks, namely to add the missing links with high likelihood of existence back to the networks. Even though large 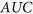 is achieved, the reconstructed networks from these existing methods are found to be very different from the true networks, especially in terms of the average *betweenness* of the predicted links. This result indicates that the missing inter-community links are seldom captured by the existing link prediction methods. To solve this problem, we propose a community-based link prediction method. Our method can effectively identify the inter-community links by slightly sacrificing the prediction accuracy. The final obtained network can thus well reproduce the structural and dynamical properties of the true network.

## Results

We consider an undirected network 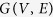 where 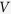 is the set of nodes and 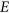 is the set of links. In link prediction, the original links 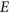 are first randomly divided into two parts: the training set (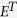) and the probe set (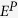). The training set contains 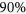 of the original links and the link prediction methods run on it. The probe set consists of the remaining 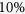 of the original links (The results of other division ratios are shown in SI). The probe set is used to test the accuracy of the link prediction methods. The accuracy is usually measured by the 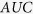 value (see the Methods section for details), the higher the better. Besides accuracy, we consider also whether the link prediction methods can effectively recover the structural properties of the original network. Normally, the link prediction methods predict missing links by assigning each unconnected node pair a score which estimates the likelihood for each node pair to have a missing link between them. An accurate link prediction method will assign high score to the true missing links and low score to the nonexistent links. Unfortunately, for most of the existing link prediction methods, there is no obvious score gap between the true missing links and nonexistent links. Therefore, in order to reconstruct the network, one has to assume that the number of true missing links 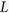 is roughly known. In this fashion, one can add 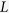 top-ranking links in the link prediction methods to the observed network to reconstruct the predicted network. The approach is widely used in the literature^38^,^39^. Consistent with the previous works, we also assume that we know roughly the total number of true missing links. The 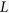 node pairs (*L* = |*E*^*P*^|) with the highest score (denoted as the “predicted links”) will be added to the training set 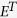 to obtain the reconstructed network *G*′(*V*, *E*′). A well-performed link prediction method should not only aim at achieving a high 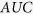 value, but also make the structural properties of *G*′(*V*, *E*′) close to *G*(*V*, *E*).

**Figure 1:**
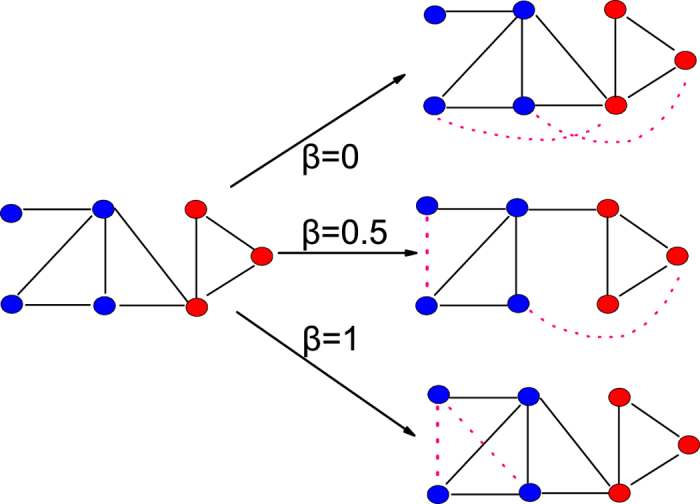
The illustration of the community-based link prediction method. The network on the left is the network consisting of the links in the training set. The nodes within one community are marked by the same color. The solid links represent the observed links and the dashed links stand for the predicted links. When *β* = 0, the inter-community missing links are ranked higher than the intra-community missing links in the prediction list. Therefore, mainly inter-community links are added to the network by the link prediction method. When *β* = 1, the intra-community missing links are ranked higher than the inter-community missing links in the prediction list, and mainly intra-community links are added to the network. When *β* = 0.05, the results are mixed, both inter- and intra-community missing links are added to the network. The similarity measure used in this toy network is CN.

In this paper, we focus on the networks with community structure. According to the definition, the nodes within a community are densely connected while the nodes across communities are much more sparsely connected. In this kind of networks, the inter-community links are in general more difficult to be predicted. Without these inter-community links, the average shortest path length of the reconstructed networks would be much higher than the original networks, and the transportation dynamics^40^ in this network would be much slower and congested in the reconstructed networks. In order to solve this problem, we propose a community-based link prediction method. We first detect the communities by using the 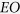 algorithm^41^ in the training set. Then the similarity scores between unconnected node pairs are computed by some classic local similarity measures (i.e. the CN or RA methods, see the Methods section for definitions). We also consider three global link prediction methods^32^,^39^,^42^, the results are similar to those of CN and RA (see Supplementary Information (SI)). A tunable parameter *β* ∈[0, 1] is proposed to combine the information of communities and node similarity for link prediction. In practice, the node pairs are classified as intra-community pairs and inter-community pairs. Within each classification, the node pairs are ranked in descending order according to the similarity measures. 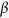 controls the probability that the intra-community node pairs ranked higher than the inter-community node pairs (see the Methods section for details). This method is inspired by ref. 43 but used here for a different goal. For convenience, when the method is combined with common neighbor similarity, it is called community-based CN method (CBCN). Similarly, it is called community-based RA method (CBRA) when it is combined with the resource allocation similarity. The illustration of the method is shown in Fig. 1. Like previous works^43^, we adopt 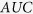 to evaluate the accuracy of the link prediction. In addition, we propose to monitor the average edge-betweenness 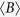 of the predicted links (calculated by adding those predicted links to the network). If the average edge-betweenness is high, more inter-community links are predicted (For the solid evidences, see SI). In fact, measuring the average betweenness of the reconstructed network is also a good evaluation metric for this issue. Despite some quantitative difference, the results are qualitatively consistent with the results when 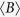 is used (see results in SI).

We first test our method in a classical artificial network: GN-benchmark network^35^ which is widely used in the research of community structure. In the GN-benchmark network, *n* = 128 nodes equally distribute in 4 communities, and each node has on average 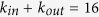 links where 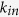 is the average number of neighbors within the same community (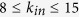) and 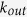 is the average number of neighbors between different communities (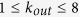). As 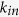 increases, the community structure of network becomes clear. Given an observed network, the obtained similarity score between nodes is deterministic if CN and RA similarity measurements are applied. However, the community detection algorithm has randomness. Therefore, there is some stochasticity in the link prediction process coming from the community detection algorithm. In this paper, we use the extremal optimization (EO) algorithm to detect communities. As stated in ref. 41, the performance of this algorithm is rather stable. Therefore, the stochasticity of the link prediction process is expected to be relatively small. We perform several times of realizations and find that the variance is much smaller than the mean value. Therefore, we mainly report the results of the mean value of different realizations.

**Figure 2:**
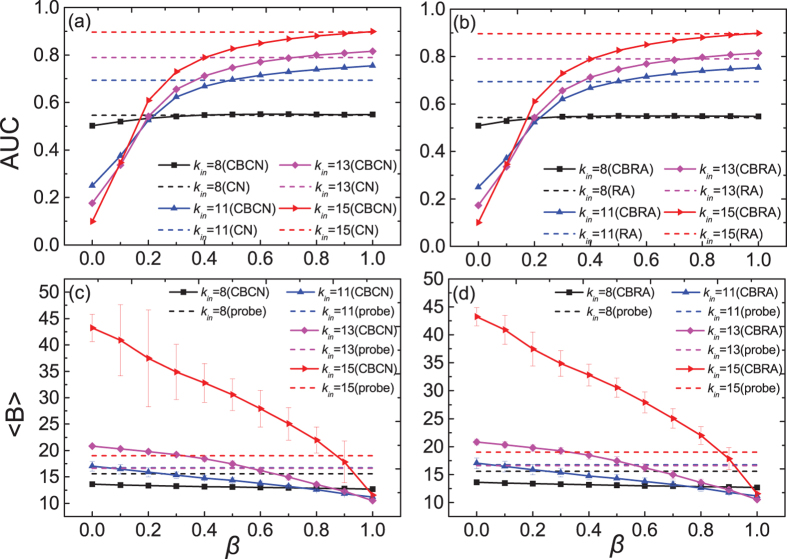
The influence of *β* on *AUC* and 〈*B*〉 in the GN-benchmark networks. (**a**–**d**) are the results of CBCN and CBRA, respectively. The solid lines are the results of the community-based link prediction methods (CBCN and CBRA) and the dashed lines are the results of the classic link prediction methods (CN and RA). The results are averaged over 100 independent realizations.

In Fig. 2, we show the dependence of 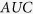 and 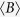 on 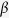 under different 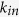. The CBCN and CBRA are used in Fig. 2(a–d), respectively. One can see that 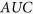 increases with 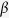, indicating that the links within the communities are easier to be predicted. The results of CBCN and CBRA are similar and the increment of 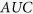 is more significant when the community structure is more obvious (i.e. larger 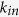). This result is consistent with a recent finding in ref. 43. In Fig. 2(a,b), the dashed lines mark the 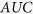 of the original CN and RA methods (without 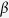 to adjust the ranking of the intra- and inter-community missing links). One can see that the 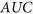 of CBCN and CBRA can be respectively higher than the 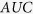 of CN and RA when 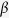 is large.

In Fig. 2(c,d), it shows that 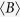 actually decreases with 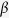. This is natural as a larger 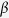 means more intra-community missing links are ranked higher, thus the predicted links are mainly within communities. In Fig. 2(c,d) the dashed lines mark the 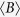 of the links in the probe set. Clearly, if one only considers 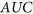, *β* = 1 is the optimal solution. However, this setting of 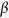 would make 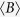 of the predicted links smaller than that of the true missing links. A good link prediction method should not only have high 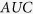 but also make 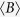 of the predicted links close to that of the true missing links. Interestingly, we observe that when 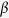 is large, a small change in 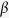 can result in a significant decrease in 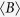 but little influence on 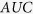. This observation indicates the possibility to adjust 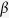 for a satisfactory results in both 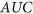 and 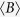.

**Figure 3:**
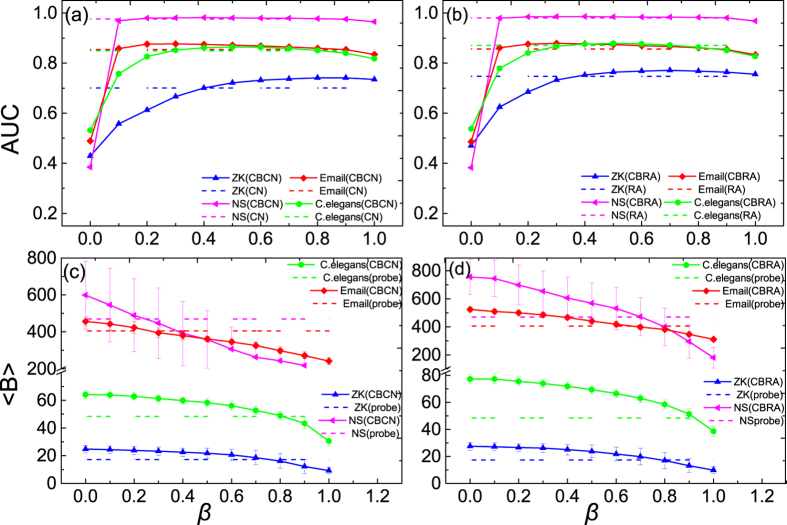
The influence of *β* on *AUC* and 〈*B*〉 in four real networks. (**a**–**d**) are the results of CBCN and CBRA, respectively. The solid lines are the results of the community-based link prediction methods (CBCN and CBRA) and the dashed lines are the results of the classic link prediction methods (CN and RA). The results are averaged over 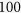 independent realizations.

We also examine our method on four real networks: *ZK* is a social network in the zahcary karate club^44^, *NS* is the largest connected component of a co-authorship network of scientists who are publishing on the topic of network science^45^, *Email* is an email network of an university built by regarding each email address as a node and linking two nodes if there is an email communication between them^46^, *C.elegans* is a neural network of the worm Caenorhadities elegans with each neuron as a node and each synapse or gap junction as a link^47^. All of these real networks are widely used in the literature and the basic structural properties of them are listed in Table 1. Here we use them to examine our methods. Figure 3 shows the performance of the community-based link prediction methods on these real networks. One can see that the results are qualitatively the same as those in the GN-benchmark networks. In these real networks, as the community structure is not as obvious as the GN-benchmark, the effect of 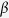 on 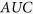 is even smaller, especially after *β* > 0.1. However, the influence of 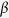 on 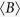 is still strong.

We denote 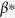 as the 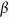 that can make 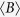 of the predicted links the same as that of the true missing links (i.e. the links in the probe set). Accordingly, the 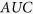 under 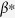 is denoted as 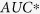. The quantitative results of 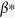 and 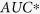 in four real networks are reported in Table 1. Clearly, the 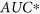 of CBCN and CBRA can still be higher than the 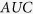 of CN and RA, respectively.

To further understand the performance of each method, we compute the number of correctly predicted inter- and intra-links and the number of inter- and intra-links in the predicted links (results are shown in SI). We find that when the existing link prediction methods are used in GN-benchmark, the number of inter-links in the predicted links is almost zero, indicating that these existing methods tend to neglect inter-links. On the contrary, CBCN and CBRA have many inter-links in the predicted links. However, if we look at the number of correctly predicted inter-links in our methods, the number is also small. This is because the inter-links are sparsely and randomly connected in GN-benchmark (i.e. almost form no triangle) and it is difficult for CBCN and CBRA to capture their similarity to other links. In real networks, however, the inter-links form more triangles than thus are easier to be predicted. We test the NS real network with clear community structure (collaboration network between network scientists). We find that CN and RA can correctly predict 17.6 and 30.0 inter-links while CBCN and CNRA can correctly predict 23.7 and 31.5 inter-links (For more detailed results in NS network, see SI). These results indicates that CBCN and CNRA can respectively outperforms CN and RA in real networks as well.

**Figure 4:**
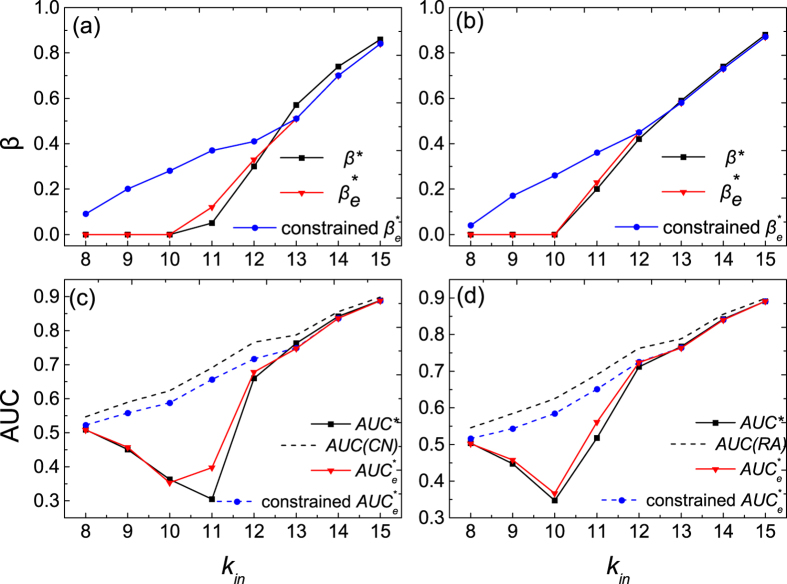
The influence of *κ*_*in*_ on β^*^ and AUC^*^ in the GN-benchmark networks. (**a**–**d**) are the results of CBCN and CBRA, respectively. The solid lines are the results of the community-based link prediction methods (CBCN and CBRA) and the dashed lines are the results of the classic link prediction methods (CN and RA). The results are averaged over 100 independent realizations.

In Fig. 4, we further investigate the influence of 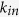 on 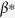 and 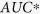 in the GN-benchmark networks. In Fig. 4(a,b), one can see that 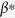 has an abrupt change after *k*_*in*_ > 10. After this value, 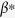 significantly increases with 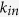. This is because when the community structure is obvious (*k*_*in*_ > 10), we don’t have to sacrifice too much 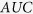 and a large 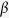 can already make 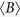 close to the true value. In Fig. 4(c,d), we show the dependence of 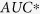 on 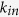. One can see that when 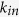 is large, 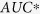 is very close to the 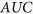 of the original CN or RA. However, when 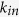 is relatively small, 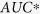 can be much smaller than 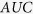 of CN or RA. This is because when 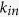 is small, 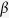 needs to be adjusted to a very small value in order to keep 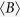 of the predicted links the same as the real links (as shown in Fig. 2). In this case, a large amount of 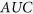 needs to be sacrificed for a higher 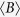.

So far, we have already shown that adjusting 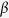 in the community-based link prediction methods can indeed help the methods predict more high-betweenness links in the networks. A natural question to ask at this point is how to choose 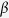 in real use. Even though 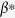 can be chosen at the value where 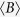 of the predicted links becomes the same as the real links. However, as 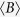 of the real links is unknown information, the above strategy seems to be an inapplicable way. To solve this problem, one has to learn the optimal 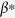 from the observed data. To mimic this process, we use a so-called threefold validation where a small part (usually 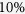 of all links) is moved from the previously introduced training set 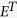 to a learning set 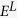 48. The threefold validation is usually used to avoid model over-fitting in machine learning. In our case, by checking at which 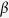 the predicted links from 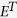 can have the same 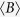 as the links in 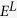, one can determine the estimated optimal parameter 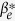.

One concern for the learning process is that the missing links may largely change the structural properties. To check this, we first conduct the community detection algorithm (EO algorithm) on the original true network and denote the obtained communities as the “true detected communities”. Then we randomly remove a fraction of links from the true network to obtain the observed network. We do again the community detection algorithm on the observed network and compute the fraction of nodes classified correctly by comparing the obtained communities with the so-called “true detected communities”. We find that the fraction of nodes classified correctly is rather high, especially when the community structure is obvious (correct rate is over 80% when *k*_*in*_ ≥ 10). Moreover, we compare 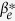 with 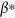 determined with 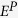 in Fig. 4(a,b). One can see that 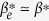 at different 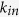.

The learned optimal parameter 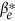 is then used to predict missing links based on 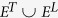 which are then compared with entries in 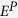 to finally measure the link prediction accuracy 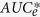. The results are shown in Fig. 4(c,d). One can see that 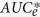 is indeed close to 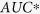. As discussed above, the 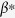 is usually too small when *k*_*in*_ < 13, which directly results in a low 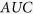 in link prediction. Therefore, we propose an additional constraint in the learning process: when determining the optimal 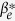 with the learning set 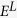, we also monitor the prediction 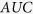 of these links in 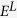 (denoted as 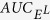). In order to make sure the optimal 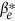 will not be too small, we assume that at most we can sacrifice 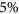 of the accuracy. Here, we define the 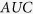 of the original method CN or RA as 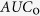. If before 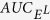 drops to 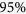 of 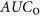, the predicted links can have the same 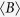 as the links in 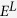, 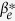 is chosen as this crossover point. If not, 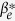 is chosen as the value where 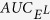 equals to 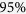 of 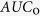. The 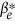 obtained in this way is denoted as “constrained 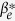
”. The results of the constrained 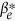 and its prediction accuracy “constrained 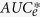
” are shown in Fig. 4 as well. So far, we have discussed three parameters: 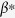, 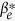 and constrained 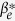. A summary of these three parameters is given in Table 2. Note that even though the amount of missing links is not known, the estimation of 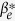 and constrained 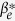 will not be influenced. This is because 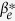 and constrained 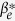 are obtained from the learning process in which the amount of links in the learning set 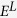 is known.

Moreover, we study whether the structural and dynamical properties of the reconstructed networks from CBCN and CBRA are truly closer to the true networks. We take into account six indices, including the average shortest path of the networks 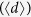, clustering coefficient (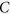)^47^, assortativity coefficient (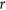)^3^, congestibility (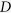)^49^, synchronizability (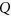)^50^ and spreading ability (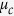)^51^. The results of different link prediction methods are listed in Table 3. The original real networks are denoted as 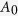. We first randomly divide the links in 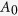 to three parts: training set 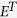 (with 80% of the links), learning set 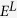 (with 10% of the links) and probe set 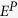 (with 10% of the links). We apply the community-based link prediction methods to compute the constrained 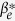 with 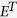 and 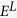. Then we do 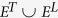 to obtain a complete 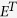. We apply the community-based link prediction methods with the constrained 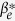 on the complete 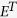. The 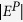 number of links with the highest link prediction score are then added to 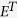 to create the reconstructed network 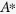. We also create the reconstructed networks with 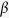 arbitrarily set as 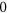 and 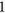, and denote these networks as 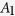 and 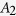, respectively. For comparison, the reconstructed networks with the traditional link prediction methods (e.g. CN and RA) are denoted as 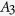. From Table 3, we can see that the reconstructed networks from the community-based link prediction methods (i.e. 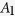, 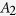 and 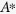) have more similar network properties to the real network 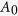 than those obtained by the traditional link prediction methods (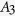). The best results sometimes appear in 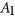 and 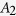. However, when 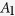 is closest to 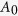, 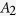 is very different from 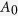, and vice versa. 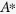 keeps a reasonable trade-off between these two methods: 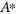 best reproduces the network properties of 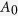 in many cases; when 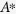 is not the best, 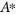 is the closest one to the best. These results confirm the importance of the parameter learning process.

Finally, we discuss the computational complexity of our method. The method is actually a combination of local link prediction algorithm and the community detection algorithm. For the local link prediction algorithm such as CN and RA, the computational complexity is 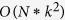 where 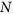 is the number of nodes and 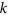 is the mean degree of the network. In this paper, we use the extremal optimization (EO) algorithm for community detection, with computational complex 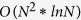. Apparently, the computational complexity in our method is mainly determined by the community detection algorithm. If the method is applied to large networks, one can choose a faster community detection algorithm, such as the method in ref. 52 with complexity 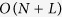 in which 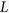 is the number of edges in the network.

## Discussion

Predicting the missing or future links is a very important research topic itself and has applications in many different domains. Although many link prediction methods have been proposed in the literature, they consider all the missing links homogeneous (i.e. all the missing links are considered equally important). In this paper, we argue that in the networks with community structure, the links connecting different communities are actually of more significance and more difficult to be predicted. We propose a community-based link prediction method which allows us to predict more missing inter-community links (with high edge-betweenness) in both artificial and real networks. The results show that our method can predict more high betweenness links without losing much link prediction accuracy. As the community-based link prediction method has a parameter to tune, we propose a learning process to determine the optimal parameter. We finally apply the community-based link prediction method to reconstruct networks. The results show that the reconstructed networks by our method have very similar network properties with the real networks.

Even though our paper tries to solve a specific problem, it points out several long-neglected important issues in link prediction research: (i) Links in the network are not with equal importance. The algorithms should give priority to those important links. (ii) Prediction results should be evaluated not only by accuracy but also by how much the predicted links can recover the properties of the true network. (iii) The parameters in the link prediction algorithms should be estimated via a learning process before applied to real prediction. These issues will encourage researchers to reconsider the existing works in link prediction and may inspire a series of more effective algorithms in the future.

In this paper, we proposes an effective method to predict the inter-community links. Compared to the existing methods which all fail to predict the inter-community links (especially when the community structure is obvious), our method has a large proportion of inter-community links in the top ranking. We admit that the improved precision of these inter-community links is not high, this is because those links have a very low probability of existing. However, by including more inter-community links in the prediction list, we manage to obtain reconstructed networks with closer topological properties to the true networks. Predicting important links in networks is a scientific problem which cannot be completely solved in one paper, it surely asks for more studies in the future. Therefore, our paper raises up some important questions for future research. The method in this paper use the classic EO community algorithm to detect communities. An interesting question would be comparing the performance of different community algorithms in helping link prediction algorithms identify inter-community links. In the networks without clear community structure, the links with high edge-betweenness are still more important than the low edge-betweenness links. In these networks, the method proposed in this paper cannot be directly applied as it relies on the community detection method. Therefore, how to predict high edge-betweenness links in networks without community structure is an important extension. Finally, our study highlights the fact that the missing links are not with equal importance. Besides betweenness, the importance of links can be measured by other properties such as degree-product, clustering coefficient, link salience^53^ etc. We hope the method in this paper will shed some light on designing methods to predict these kinds of important links in complex networks.

## Methods

### Classic link prediction algorithms

We use two representative classic link prediction algorithms in this paper: common neighbors (CN) and resource allocation (RA). After the network data is divided into the training set 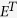 and probe set 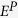, these two methods generate the predicted links by estimating the similarity values between different node pairs in 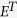. We denote the set of neighbors of node 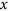 by 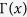.

CN simply measures the similarity between node 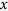 and node 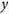 with the number of overlapped neighbors,


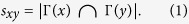


RA is a variant of CN. In RA, the weight of each common neighbor is negatively proportional to its degree. The similarity is thus computed as


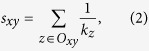


where 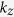 is the degree of node 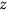 and 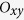 is the set of the common neighbors between 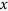 and 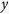. After obtaining 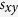 for each node pairs, the missing links is ranked by sorting 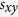 in descending order.

### Community detection

The community detection method in the paper is the EO method^41^. It detects communities by optimizing the modularity 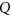 with a heuristic search. The modularity 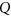 is defined as


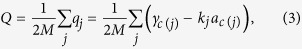


where 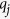 is the contribution of individual node 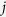 given a certain partition into communities. 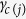 is the number of links node 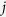 has with nodes in the same community 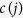, 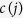 is the community which node 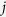 belongs to. 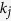 is the degree of node 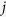 and 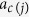 is the fraction of links that have one or two nodes inside of the community 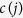. 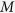 is the number of the links in the network.

### Community-based link prediction method

After computing 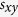, the node pairs are classified into two sets according to the community detection results: intra-community node pairs and inter-community node pairs. The node pairs in each set are ranked according to 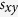 in descending order. The ranking list in intra-community node pairs is denoted as *R*_inter_ and the ranking list in inter-community node pairs is denoted as *R*_inter_. The parameter 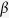 is used when *R*_inter_ and *R*_inter_ are combined. Initially, 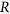 is empty. The node pairs are then moved from *R*_inter_ and *R*_inter_ to 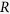 one by one from top to bottom. In each step, *R*_inter_ is picked with probability 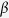 and 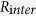 is picked with probability 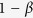. For instance, if there is already 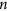 node pairs in 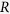 and in next step *R*_inter_ is picked, highest ranked node pair in *R*_inter_ is removed and placed in the 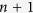 position in 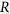. Note that the ranking list *R*_inter_ and *R*_inter_ become shorter and shorter while the ranking list 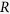 becomes longer and longer. The procedure is terminated if both *R*_inter_ and *R*_inter_ are empty.

### Result evaluation

The results of the link prediction are evaluated by 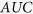 and 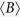. 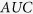 (area under the 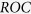 curve) is a way to quantify the accuracy of prediction algorithms^54^. At each time, we randomly select a nonexisting link in the original network and a link in the probe set to compare their positions in 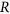. After *n* times of comparison, there are *n*′ times the probe set links have a higher rank and *n*″ times the probe set links have the same rank as the nonexisting links, then the 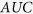 value is


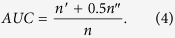


Besides 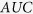, we considered another important metric called *Precision*. It is defined as the fraction of correctly predicted links in the top-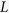 ranking list. Here, 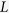 is set as the total number of missing links. The results are shown in SI. Despite some quantitative difference, the results of precision are qualitatively consistent with that of 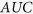 (i.e. prediction accuracy increases with 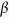).


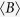
 is defined as the average betweenness of the predicted links when they are added to the networks. The predicted links are just 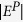 number of top ranking links in 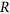. The betweenness of a link 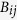 is defined as the ratio of the shortest paths which pass through the edge 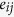 among all the shortest paths in the network,


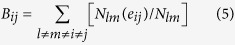



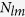
 is the number of shortest routes between node 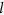 and 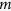, 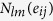 is the number of the shortest paths between node 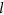 and 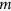 which pass through the edge 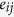.

## Additional Information

**How to cite this article**: Zhang, P. *et al.* The reconstruction of complex networks with community structure. *Sci. Rep.* **5**, 17287; doi: 10.1038/srep17287 (2015).

## Supplementary Material

Supplementary Information

## Figures and Tables

**Table 1:** Basic structural properties (network size *N*, edge number *E*, average degree **〈** *k* **〉**) of the real networks, and *β* ^*^ of CBCN and CBRA and *AUC* of the four methods when applied to these networks (*AUC* of CBCN and CBRA is obtained when *β* = *β* ^*^).

Network	*N*	*E*	〈*k*〉	β^*^		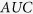			
				*CBCA*	*CBRA*	*CBCN*	*CN*	*CBRA*	*RA*
ZK	34	78	4.59	0.72	0.82	0.724	0.695	0.752	0.734
NS	379	914	4.82	0.35	0.74	0.982	0.978	0.984	0.981
email	1133	5451	9.62	0.26	0.63	0.875	0.855	0.873	0.856
C.elegans	297	2148	14.5	0.80	0.93	0.852	0.850	0.847	0.870

The results are averaged over 100 independent realizations.

**Table 2:** The description of the parameters *β* ^*^, 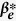 and Constrained 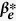 .

Parameter	Data division	Description
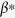	10% 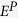 , 90% 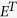	determined when 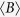 of the top-10% ranking links equals to 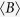 in 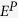
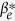	10% 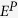 , 10% 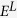 , 80% 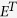	determined when 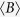 of the top-10% ranking links equals to 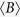 in 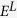
Constrained 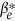	10% 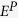 , 10% 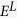 , 80% 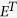	(1) If 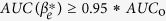 in 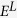 , constrained 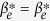 . (2) If 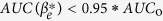 in 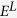 , constrained 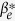 is set as the 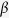 which makes 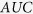 equal to 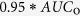 in 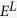

Here, *AUC*_o_ means the *AUC* value of the original link prediction methods such as CN or RA.

**Table 3:** The properties of the reconstructed networks when different link prediction methods are applied.

Net	properties	*A*_0_	*CBCN*			*CN*	*CBRA*			*RA*
			*A*_1_	A^*^	*A*_2_	*A*_3_	*A*_1_	A^*^	*A*_2_	*A*_3_
ZK	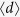	2.41	2.28	**2.37**	2.45	2.58	2.25	**2.40**	2.46	2.49
	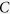	0.571	**0.583**	0.595	0.550	0.612	0.611	0.623	**0.584**	0.668
	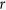	−0.476	−0.369	−0.388	−**0.438**	−0.193	−0.389	−0.430	**−0.469**	−0.204
	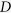	462	502	**466**	466	469	492	466	**460**	469
	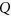	38.7	57.6	42.5	**40.7**	48.6	52.9	42.4	**40.9**	49.2
	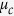	7.77	8.64	**7.77**	7.54	8.66	8.41	**7.79**	7.46	8.80
NS	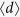	6.04	**6.12**	6.24	6.42	6.80	5.83	**6.16**	6.42	7.12
	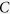	0.741	0.654	0.668	0.667	**0.685**	0.694	**0.728**	0.713	0.724
	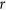	−0.0817	**0.0037**	0.0183	0.0335	0.0670	−0.1004	−**0.0834**	−0.0712	0.0485
	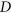	5.7·10^4^	**5.7·10**^**4**^	6.1·10^4^	6.2·10^4^	5.9·10^4^	**5.7·10**^**4**^	6.3·10^4^	6.0·10^4^	5.7·10^4^
	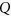	2305	**2747**	3421	2861	3447	**2458**	3128	2787	3150
	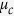	8.02	8.73	8.70	**8.15**	9.21	8.38	**8.07**	7.76	8.74
Email	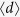	3.61	**3.63**	3.65	3.68	3.75	3.57	**3.61**	3.63	3.71
	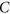	0.220	**0.223**	0.232	0.238	0.233	0.327	0.358	**0.314**	0.339
	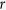	0.0782	0.163	0.165	**0.150**	0.238	0.0753	0.0756	**0.0782**	0.212
	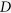	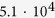	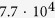	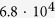	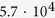	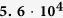	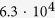	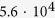	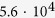	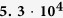
	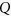	217	331	307	**304**	372	235	**205**	262	273
	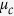	18.7	21.7	21.5	**20.5**	21.6	19.7	19.4	**19.1**	19.9
C.elegans	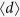	2.45	**2.44**	2.47	2.49	2.53	2.40	**2.47**	2.48	2.56
	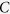	0.292	0.333	0.351	**0.333**	0.349	**0.369**	0.385	0.369	0.384
	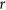	−0.163	−0.113	−0.0980	−**0.135**	−0.0405	−0.130	−0.129	−**0.162**	−0.0428
	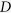	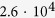	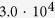	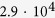	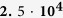	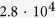	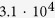	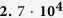	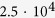	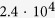
	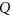	159	176	**168**	148	195	185	**163**	151	217
	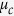	26.1	29.7	28.2	**26.9**	31.5	29.7	27.3	**26.4**	32.1

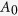
 represents the original networks, and 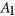, 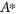, 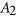 stand for the reconstructed networks, when *β* = 0, *β* = constrained 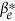, *β* = 1 respectively. 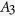 is the reconstructed networks of the traditional methods CN and RA. 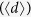, 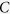, 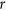, 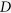, 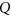, 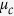 in turn, represent the average shortest path, the clustering coefficient, the assortativity coefficient, congestability, synchronizability and spreading ability of the networks. We highlight the values that are closest to the original networks in bold font. The results are averaged over 100 independent realizations.
