# Supplementary material for: The reconstruction of complex networks with community structure
Source: Sci Rep. 2015 Dec 1;5:17287. doi: 10.1038/srep17287 (PMC4664866; doi:10.1038/srep17287)
Supplement: Supplementary Information [file srep17287-s1.pdf]

Supplementary Information
The reconstruction of complex networks with community structure

Peng Zhang, Futian Wang, Xiang Wang, An Zeng and Jinghua Xiao

I. THE RELATION BETWEEN $\langle B\rangle$ AND INTER-LINK RATIO

We tune the parameter β and record the average edge-betweenness of the predicted links ($\langle B\rangle$) and the fraction of inter-community links in the predicted links (inter-link ratio) under different β. We then plot $\langle B\rangle$ versus inter-link ratio in Fig. S1. One can see that when the average-betweenness of the predicted links is high, indeed more inter-links are predicted.

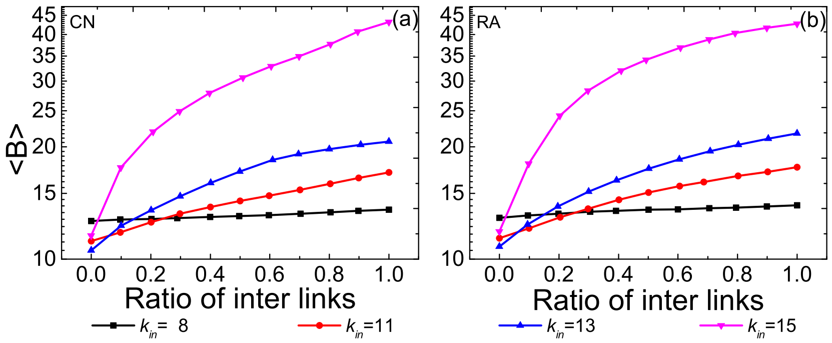

Fig. S1, the influence of β on AUC and $\langle B\rangle$ in GN-benchmark networks when Katz and SPM methods are applied. The results are averaged over 100 independent realizations.

II. RESULTS OF TWO GLOBAL LINK PREDICTION METHODS

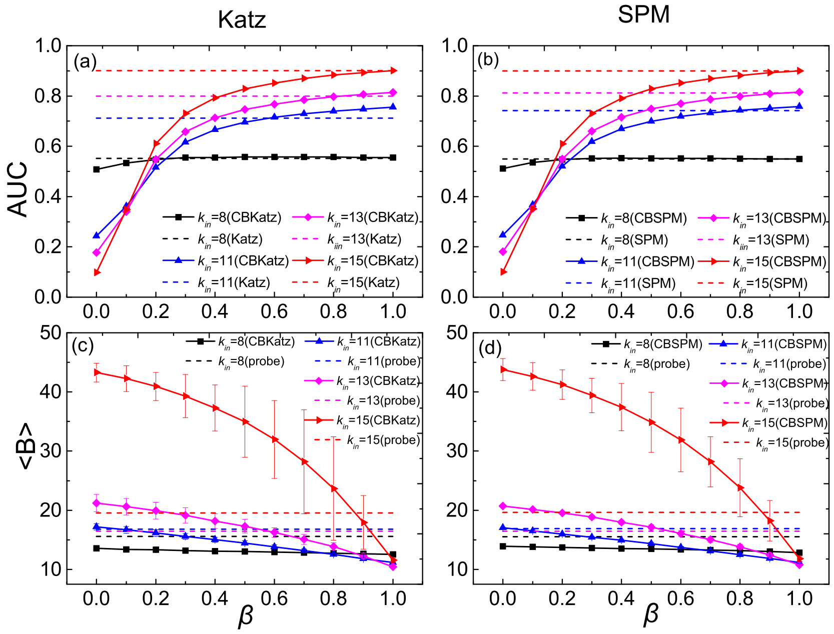

Fig. S2, the influence of β on AUC and $\langle B\rangle$ in GN-benchmark networks when Katz and SPM methods are applied. The results are averaged over 100 independent realizations.

We tested two global link prediction algorithms, the Katz [1], SPM [2] and LR [3] methods, in the GN benchmark.

Table. S1, the number of correctly predicted inter- and intra-links (the number on the left in each column), and the number of inter- and intra- links (the number on the right in each column) for each method. The original networks are GN-benchmark networks with different k_{in}. The results are averaged over 100 independent realizations.

	the precision of intra-links				the precision of inter-links			
Methods	12	13	14	15	12	13	14	15
CN	12.08/199.2	12.96/206	16.1/208	16.04/205.8	0/0.48	0/0	0/0	0/0
RA	12.62/197.8	6.24/104.8	16.04/207.7	17.72/205.9	0/2.16	0/0.08	0/0	0/0
Katz	11.48/199.4	12.94/206	16.5/208	15.82/205.8	0/0.58	0/0	0/0	0/0.18
SPM	12.64/198.3	14.86/205.3	16.32/207.9	14.48/206	0/1.66	0/0.7	0/0	0/0
LR	10.8/200	12.4/206	15.6/208	13.2/206	0/0	0/0	0/0	0/0
CBCN	5.26/80.52	6.24/104.8	9.1/120.1	13.7/173.7	0.18/119.5	0.08/101.2	0/87.88	0.06/32.32
CBRA	5.68/90.04	9.08/143.6	11.5/153.5	14.64/179	0.3/110	0.06/62.4	0.04/54.46	0.02/27

Table. S2, the results of CN, CBCN, RA and CBRA link prediction methods in the NS network. The predicted inter-links are the inter-community links in the top-L ranking list, and the correct inter-links are the true missing inter-community links among the predicted inter-links. Correct rate=number of correct inter-links/number of predicted inter-links. The results are averaged over 100 independent realizations.

Methods	CN	CBCN	RA	CBRA
the number of correct intra-links	77.1	50.9	74.5	73.5
the number of predicted intra-links	129.9	62.8	129.5	129.0
correct rate of intra-links	59%	81%	58%	57%
the number of correct inter-links	17.6	23.7	30.0	31.5
the number of predicted inter-links	54.1	121.2	54.5	55.0
correct rate of inter-links	32%	19%	55%	58%

The results of Katz, SPM and LR are shown in Fig. S2. One can see that the results are consistent with the local similarity methods (CN and RA). Specifically, when these two global methods are combined with the community detection method, tuning the parameter β can improve the ability for predicting high betweenness links.

III. THE PREDICTION ACCURACY ON INTER- AND INTRA-LINKS

For each method, we compute the number of correctly predicted inter- and intra-links and the number of inter- and intra-links in the predicted links. We report the results in table S1. One can see that, when the local methods (CN, RA) and global methods (Katz, SPM, LR) are used, the number of inter-links in the predicted links is almost zero, indicating that these existing methods tend to neglect inter-links. On the contrary, our methods, CBCN and CBRA, have many inter-links in the predicted links. Having these inter-links in the predicted links is very meaningful in network reconstruction. After adding them to the observed network, the connectivity between communities can be increased, resulting in closer network properties to the true network.

However, if we look at the number of correctly predicted inter-links in our methods, the number is also small. This is because the inter-links are sparsely and randomly connected in GN-benchmark (i.e. almost form no triangle) and it is difficult for CBCN and CBRA to capture their similarity to other links. In real networks, however, the inter-links form more triangles and thus are easier to be predicted. We test the NS real network with clear community structure (collaboration network between network scientists). We list the results of different link prediction methods in table S2. One can see that indeed CBCN and CBRA can correctly predict more inter-community links than CN and RA, respectively. However, there are also more inter-community links in the prediction lists of CBCN and CBRA than CN and RA. Therefore, the correct rates of CBCN and CBRA are not always higher than CN and RA (correct rate=number of correct inter-links/number of predicted inter-links). Moreover, as CBCN and CBRA aim to predict more inter-community links, they predict fewer intra-community links than CN and RA, respectively (see also table S2).

We then define the prediction accuracy of inter-links as the number of correctly predicted inter-links over the number of inter-links in the predicted links. We then study the dependence of the prediction accuracy of inter-links on β in GN-benchmark networks, as shown in Fig. S3. One can see that the prediction accuracy of inter-links is zero for the CN and RA methods (expect when $k_{in} = 8$ where no community structure). For the CBCN and CBRA, the prediction accuracy of inter-links is nonzero but low. As we discussed above, this is because the inter-links are sparsely and

randomly connected in GN-benchmark and it is difficult for CBCN and CBRA to capture their similarity to other links. We also test the NS real network with clear community structure (collaboration network between network scientists) in Fig. S4. One can see that the prediction accuracy of inter-links in this case is much higher. This is because the inter-links in real networks form some triangle structures, which allows link prediction methods to estimate the similarity between them and other links.

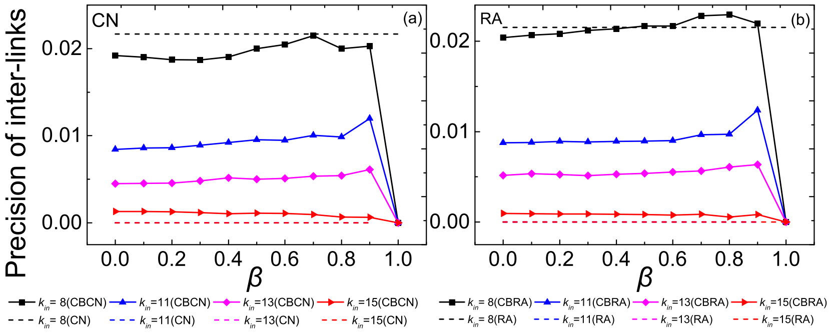

Fig. S3, the dependence of the prediction accuracy of inter-links on β in GN-benchmark networks. The results are averaged over 100 independent realizations.

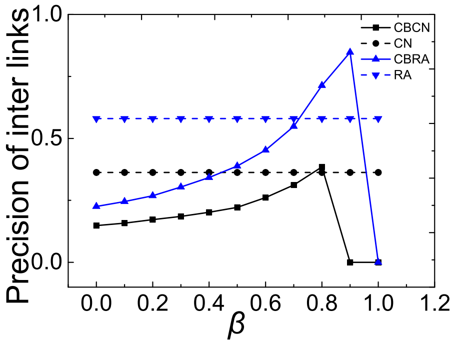

Fig. S4, the dependence of the prediction accuracy of inter-links on β in NS network. The results are averaged over 100 independent realizations.

IV. AVERAGE BETWEENNESS OF THE RECONSTRUCTED NETWORKS

In a network with community structure, the inter-community links tend to have high edge betweenness. If one can predict these high betweenness links, generally more inter-community links are predicted. In fact, measuring the average betweenness of the reconstructed network is also a good evaluation metric for this issue. Accordingly, we compute the average betweenness of the reconstructed networks and present the results in Fig. S5. One can see that when the traditional link prediction methods are used, the average beweenness of the reconstructed networks is high, indicating that there are only a few links between communities carrying most of the shortest paths. With $\beta < 1$, more inter-community links are predicted. Therefore, there are more links between communities to share the shortest paths in the reconstructed network, resulting in a smaller average betweenness of the reconstructed network. The results show that tuning β can indeed better reproduce the property of the true network (i.e. the average betweenness of the reconstructed network is the same as the true network).

β^* can also be determined by checking at which β the reconstructed network has the same average betweenness as the original network. The results are shown in Fig. S6. When k_{in} is small (i.e. the community structure is not obvious), the β^* obtained in both ways is zero. When k_{in} is large (i.e. the community structure is obvious), β^* obtained in these two methods is still close to each other (with the new β^* slightly lower than the β^* in the paper).

V. RESULTS OF PRECISION

We use the *Precision* to measure the accuracy of the predicted links and plot the precision versus β in GN-benchmark networks. The precision is defined as the fraction of correctly predicted links in the top-E ranking list. Here, E is

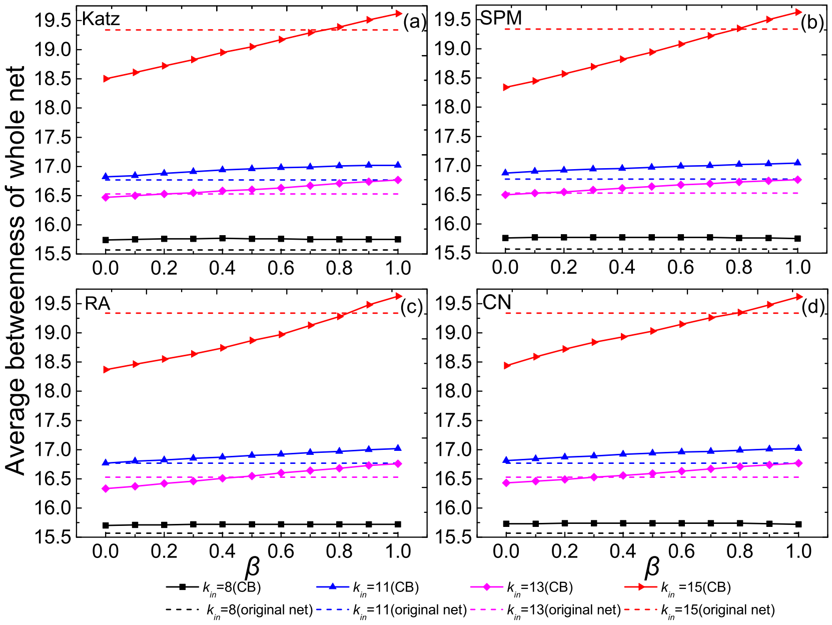

Fig. S5, the average betweenness of the reconstructed network under different parameter β. The original network is a GN-benchmark model. The results are averaged over 100 independent realizations. In the legend, CB means the new method that combines the existing link prediction method (in each subplot) with the community detection method (EO algorithm).

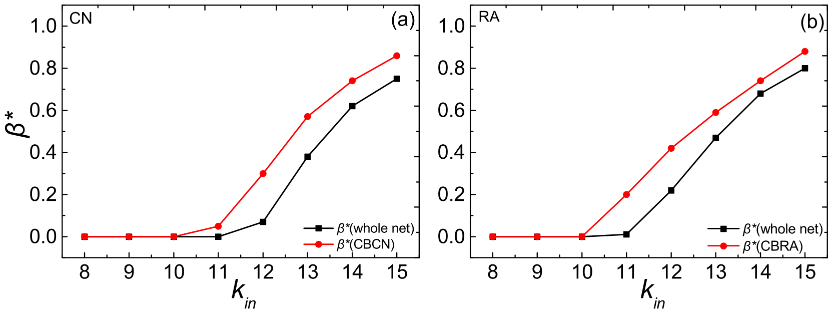

Fig. S6, the obtained β^* of two methods versus k_{in} of the GN-benchmark networks. The "whole net" means that β^* is obtained when the reconstructed network has the same average betweenness as the original network. The "CBCN" means that β^* is obtained when the average betweenness of the predicted links are the same as that of the true missing links. The results are averaged over 100 independent realizations.

set as the total number of missing links. One can see from Fig. S7 that the trends are consistent with AUC (i.e. prediction accuracy increases with β).

VI. RESULTS OF DIFFERENT DIVISION RATIO

Here, we present the results under different fraction of removed links, as shown in Fig. S8. One can see that when the training set has more than 50% links, the constrained β_e^* and AUC are rather stable. However, when the amount of links in the training set is smaller than 50%, the constrained β_e^* and AUC are substantially changed. This is because the community structure becomes vague if the links in the training set is too sparse. For the average betweenness of the predicted links, it keeps monotonously increasing when the link ratio in the training set becomes small. This is because when the link ratio in the training set is small, more shortest paths need to go through the predicted links that are added to the network.

[1] Katz, L. A new status index derived from sociometric analysis. *Psychometrika* **18**, 39 (1953).

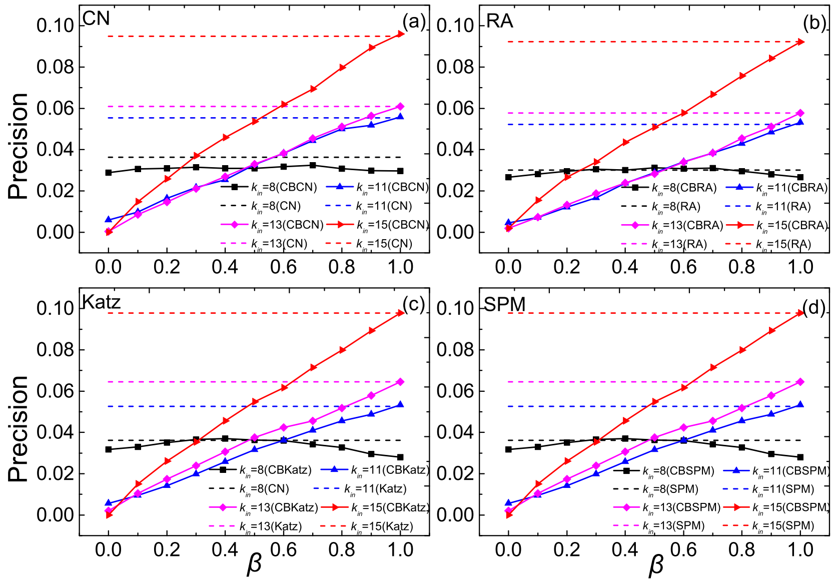

Fig. S7, the precision versus β in GN-benchmark when different link prediction methods are applied. The results are averaged over 100 independent realizations.

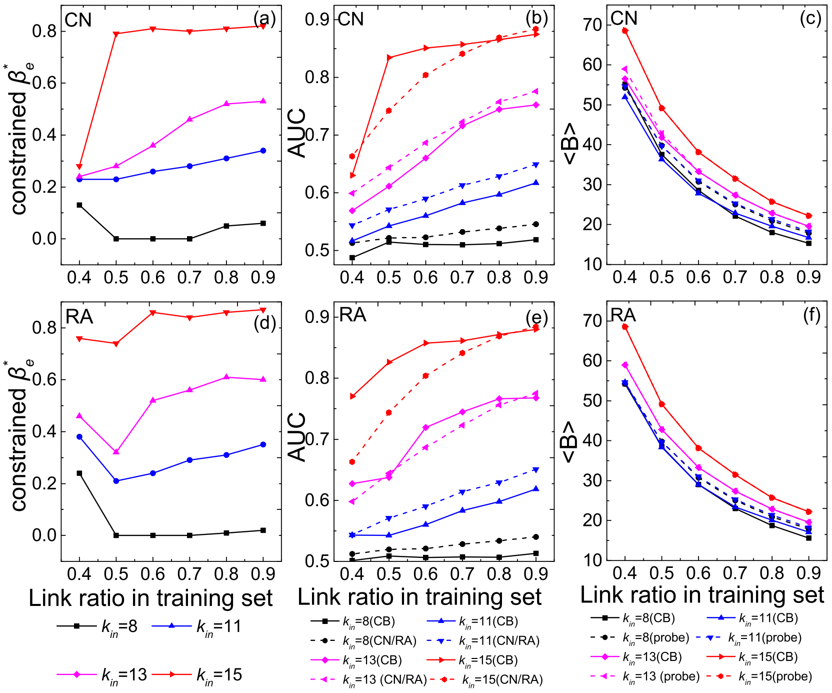

Fig. S8, the constrained β_e^*, AUC and $\langle B\rangle$ under different fraction of removed links. The networks are GN-benchmark networks. The results are obtained by averaging 100 independent realizations. In the legend, CB means the new method that combines the existing link prediction method (in each subplot) with the community detection method (EO algorithm).

[2] Lü, L., Pan, L., Zhou, T., Y.-C. Zhang & H. E. Stanley, Toward link predictability of complex networks. *Proc. Natl. Acad. Sci. U.S.A.* **112**, 2325 (2015).

[3] Guimerà, R. & Sales-Pardo, M. Missing and spurious interactions and the reconstruction of complex networks. *Proc. Natl. Acad. Sci. U.S.A.* **106**, 22073 (2009).
